# Editorial: Lipid influence on lung immune-structural cell crosstalk: implications for respiratory health

**DOI:** 10.3389/fimmu.2026.1852625

**Published:** 2026-05-06

**Authors:** Benoit Allard

**Affiliations:** Université de la Réunion, INSERM, UMR 1188 Diabète Athérothrombose Thérapies Réunion Océan Indien (DéTROI), Sainte-Pierre, France

**Keywords:** innate immune cells, lipid mediators, lipids, lipoproteins, lung structural cells, resolving inflammation, respiratory infection, surfactant

The lung is not only a gas-exchange organ but also a highly specialized lipid-rich immunological interface. The COVID-19 era, together with continued work on bacterial pathogenesis and inflammation resolution, has sharpened the view that lipids are not passive membrane components or surfactant constituents alone. They are signaling molecules, structural regulators, metabolic substrates, and therapeutic candidates that shape the dialogue between epithelial, endothelial, stromal, and immune cells in the respiratory tract. The articles assembled in this Research Topic capture this idea from complementary angles and, importantly, recent studies published since the launch of the Topic further strengthen the notion that pulmonary lipid biology is central to respiratory homeostasis, infection, and repair.

The contribution by Ji et al. drew attention to pulmonary surfactant lipids as regulators of innate immunity, extending the classical biophysical view of surfactant. Their review emphasized that phosphatidylcholine species, phosphatidylglycerol (PG), and phosphatidylinositol (PI) do more than stabilize alveoli: they influence pathogen sensing, inflammatory tone, and epithelial protection (1). This conceptual advance has aged well. More recent work has consolidated the idea that the minor anionic surfactant phospholipids PG and PI act as broad antagonists of multiple Toll-like receptor pathways and can inhibit infection by several respiratory RNA viruses, including SARS-CoV-2 and its variants (1). These observations support a model in which the air-liquid interface is not simply coated by surfactant, but actively immunoregulated by lipid species that tune the activation threshold of macrophages and epithelial cells. In that sense, surfactant lipids emerge as endogenous gatekeepers of immune-structural cell crosstalk rather than inert barriers. Recent human data also extend the translational relevance of this surfactant-centered perspective. In 2025, Kokelj et al. reported that even mild to moderate COVID-19 is associated with persistent alterations in small-airway surfactant phospholipids, including increased oxidized phospholipids and sustained evidence of small-airway dysfunction (2). These findings are notable for two reasons. First, they indicate that surfactant disturbance is not restricted to severe acute respiratory distress but can be detected in milder disease. Second, they suggest that lipid remodeling may persist beyond the acute phase and therefore contribute to prolonged epithelial dysfunction or altered repair programs. Together with the review by Ji et al., these findings reinforce the idea that surfactant composition is both a readout and a regulator of local inflammatory status.

The original article by Dubuc et al. addressed a second major axis of the Topic: inflammatory lipid mediators in viral pneumonia. By combining lung lipidomics, cytokine profiling, histopathology, and transcriptomics in SARS-CoV-2-infected K18-hACE2 mice, the authors showed that viral pneumonia is accompanied by a selective remodeling of pulmonary lipid mediator networks rather than a uniform increase in all eicosanoids. This remains an important message. It argues against oversimplified views of the “lipid storm” and instead points toward context-specific mediator programs that evolve with time, tissue compartment, and host species. Recent studies have further refined this concept by adding spatial and cellular resolution. Single-cell and spatial analyses of SARS-CoV-2-infected lungs have identified temporally organized macrophage programs linked to viral clearance and subsequent resolution, including monocyte-derived Slamf9-positive macrophages that coordinate with neutrophils and later transition toward reparative states (3). Although not focused exclusively on lipid mediators, these studies are highly relevant to this Research Topic because they provide the missing cellular framework into which lipidomics could now be integrated. The next step for the field will be to connect specific lipid mediator signatures to defined structural and immune cell niches - alveolar type II cells, resident and recruited macrophages, endothelial microdomains, and sites of thromboinflammation - rather than treating the inflamed lung as a single biochemical compartment.

The mini-review by Constantino-Teles et al. provided a complementary and highly valuable perspective by shifting attention from host-derived lipids to the lipid-centered virulence strategies of *Pseudomonas aeruginosa*. Their synthesis of the literature on lipopolysaccharide remodeling, rhamnolipids, outer membrane vesicles, and phospholipases emphasized that respiratory infection by *P. aeruginosa* can be understood as a form of lipid warfare. This framing remains compelling. It places bacterial lipids and lipid-modifying enzymes at the center of epithelial injury, immune evasion, biofilm resilience, and dysregulated host inflammation. Recent work strengthens this view. In 2024, rhamnolipid micelles were shown to be directly cytotoxic, with particularly strong effects on macrophages and additional membrane damage in human lung epithelial cells (4). This is important because it moves rhamnolipids from the category of generic biosurfactants to that of precise mediators of host-cell injury and altered cell-cell communication. The consequence is not only tissue damage but also distortion of the immune-structural dialogue on which effective host defense depends. Lipid-targeted anti-virulence strategies therefore remain especially attractive in *P. aeruginosa* infection: they may reduce damage and restore productive crosstalk without relying exclusively on bactericidal pressure.

The clinical study by Sánchez-García et al. completes the Research Topic by focusing on the resolution phase of inflammation. Their finding that lower circulating lipoxin A4 (LXA4) associates with fatal COVID-19 outcomes highlights a key principle: respiratory pathology is determined not only by the magnitude of inflammatory activation but also by the adequacy of endogenous resolution programs. This idea aligns with a broad body of work on specialized pro-resolving mediators (SPMs), but their study is particularly valuable because it translates the concept to a clinically accessible biomarker in a well-defined human cohort. The field has advanced rapidly in this direction. A comprehensive 2025 review by Serhan and Levy summarized evidence that lipoxins, resolvins, protectins, and maresins regulate leukocyte trafficking, efferocytosis, epithelial repair, and the return to homeostasis across the respiratory system (5). In parallel, preclinical therapeutic studies have become more concrete. Li et al. showed that pulmonary delivery of SPM-based nanotherapeutics attenuates experimental pulmonary fibrosis, providing a translational example of how resolution biology can be engineered into inhaled interventions (6). More recently, exogenous maresin 1 and LXA4 were reported to reduce acute lung inflammation and improve survival in severe murine chemical lung injury (7). Taken together, these findings broaden the significance of the work by Sánchez-García et al.: LXA4 is not only a candidate biomarker of disease severity, but part of an expanding therapeutic logic in which restoring resolution may limit tissue injury while preserving host defense.

Viewed together, the articles in this Research Topic reveal a coherent conceptual map. Surfactant lipids modulate the frontline conversation between epithelial cells and innate immune sentinels. Inflammatory and pro-resolving lipid mediators shape the amplitude, quality, and termination of pulmonary inflammation. Pathogens such as *P. aeruginosa* hijack or damage these circuits using their own lipid arsenal. Clinical outcome, in turn, depends not only on pathogen burden or cytokine release but on whether tissue-protective and pro-resolving lipid programs are maintained. Several priorities now emerge for the field. First, pulmonary lipid biology must be studied with stronger spatial and cellular precision. Bulk bronchoalveolar lavage or whole-lung homogenates remain informative, but they average out functionally distinct niches. Integrating targeted and untargeted lipidomics with spatial transcriptomics, imaging mass spectrometry, and single-cell profiling should allow direct mapping of lipid mediator programs to epithelial, endothelial, fibroblast, and myeloid cell states. Second, future studies should better connect mechanism to compartment. The upper and lower airways, alveolar space, vascular interface, and biofilm-rich microenvironments are not interchangeable lipid habitats. Third, translational efforts should move beyond descriptive lipid signatures toward intervention-ready questions: which lipid species can serve as robust compartment-specific biomarkers, which receptors and biosynthetic nodes are druggable, and when in the disease course should lipid-directed therapies be deployed? The translational implications are broad. One avenue is the development of multiparametric biomarker panels that combine pro-inflammatory eicosanoids, oxidized phospholipids, and SPMs to stratify patients more precisely than cytokines alone. A second is the rational revisiting of surfactant-based therapy, not merely as replacement for impaired mechanics but as immunomodulation at the epithelial surface. A third is the expansion of resolution pharmacology, including stable SPM analogues, receptor agonists, and inhaled delivery platforms. A fourth is anti-virulence targeting of bacterial lipid pathways, particularly in chronic or multidrug-resistant *P. aeruginosa* infection, where preserving lung architecture is as critical as reducing bacterial load.

This Research Topic was conceived to explore how lipids influence communication between lung structural and immune cells. The assembled articles achieve that goal and, importantly, remain timely. Since their publication, newer data have not diluted their message; they have deepened it. Lipids are emerging as organizers of pulmonary ecosystems - molecules that couple mechanics to immunity, infection to repair, and local cell states to clinical outcome. Understanding this language of lipids will be essential for developing the next generation of respiratory biomarkers and therapies.

We thank all authors, reviewers, and the editorial team for contributing to this Research Topic. We hope this Research Topic will continue to stimulate interdisciplinary work across pulmonary biology, lipidomics, immunology, microbiology, and translational medicine, and will help position lipid-centered immune-structural crosstalk as a defining framework for respiratory research.
